# The association between serum alanine aminotransferase and hypertension: A national based cross-sectional analysis among over 21 million Chinese adults

**DOI:** 10.1186/s12872-021-01948-0

**Published:** 2021-03-19

**Authors:** Jiajing Jia, Ying Yang, Fangchao Liu, Minjin Zhang, Qin Xu, Tonglei Guo, Long Wang, Zuoqi Peng, Yuan He, Yuanyuan Wang, Ya Zhang, Hongguang Zhang, Haiping Shen, Yiping Zhang, Donghai Yan, Xu Ma, Puhong Zhang

**Affiliations:** 1 Graduate School of Peking, Union Medical College, Beijing, People’s Republic of China; 2 National Research Institute for Family Planning, Da Huisi Road, 12#, Beijing, People’s Republic of China; 3 National Human Genetic Resources Center, Beijing, People’s Republic of China; 4 China DOHaD Research Center, Beijing, People’s Republic of China; 5 Department of Epidemiology, Fuwai Hospital, Beijing, People’s Republic of China; 6 National Center for Cardiovascular Disease, Beijing, People’s Republic of China; 7 China Academy of Medical Sciences and Peking Union Medical College, Beijing, People’s Republic of China; 8 School of Public Health, Institute of Epidemiology and Statistics, Lanzhou University, Lanzhou, People’s Republic of China; 9 Department of Maternal and Child Health, National Health and Family Planning Commission of the PRC, Beijing, People’s Republic of China; 10 Diabetes Research Program, The George Institute for Global Health At Peking University Health Science Center, Zhi Chun Road, 6#, Beijing, People’s Republic of China

**Keywords:** Alanine aminotransferase, Hypertension, Blood pressure, China, Cross-sectional study

## Abstract

**Background:**

Inconsistent results were found in the association between serum alanine aminotransferase (ALT) and hypertension among population-based studies. This study evaluated the association between ALT and hypertension among Chinese reproductive-age population by utilizing registration data from National Free Pre-pregnancy Checkups Project in 2016–2017.

**Methods:**

The 21,103,790 registered participants were eligible for analysis, including women who were 20–49 years old and men who were 20–59 years old with available data for ALT and blood pressure (BP). Logistic regression was conducted to estimate odds ratio (OR) for the association between ALT and hypertension as a binary outcome. Linear regression was used to examine the association between ALT and BP as a continuous outcome.

**Results:**

In total, 4.21% of the participants were hypertensive, and 11.67% had elevated ALT (> 40 U/L). Hypertension prevalence was 3.63% and 8.56% among participants with normal and elevated ALT levels. A strong linear relationship was found between serum ALT levels and the odds of hypertension after adjustment for potential confounders. The multivariable-adjusted ORs for hypertension were 1, 1.22 (1.21, 1.22), 1.67 (1.65 1.68), 1.78 (1.76, 1.80), and 1.92 (1.90, 1.94) in participants with ALT levels of ≤ 20, 20.01–40, 40.01–60, 60.01–80, and > 80 U/L, respectively. Systolic and diastolic BPs rose by 1.83 and 1.20 mmHg on average, for each 20 U/L increase in ALT (*P *_*for trend*_ < 0.001). The association was consistent among subgroups and tended to be stronger among populations who are overweight (body mass index ≥ 24 kg/m^2^) (χ^2^ = 52,228, *P* < 0.001), alcohol drinking (χ^2^ = 100,730, *P* < 0.001) and cigarette smoking (χ^2^ = 105,347, *P* < 0.001).

**Conclusions:**

Our cross-sectional analysis suggested a linear association between serum ALT and hypertension or BP, which indicated that abnormal liver metabolism marked by elevated serum ALT could play a role in hypertension or elevated BP condition.

## Background

Serum alanine aminotransferase (ALT), a sensitive and simple indicator for liver injury in clinical practice [1], was demonstrated to be closely related to obesity [2], fatty liver [3], type 2 diabetes mellitus [4], and metabolic syndrome (MetS) [5–7] in recent decades. With the increasing prevalence of these metabolic disorders, the association between ALT and metabolic diseases has attracted considerable interest. Moreover, elevated serum ALT is a new biomarker in predicting the incidence of cardiovascular disease (CVD) and poor cardiac risk profile [8–10]. As a risk factor of CVD, hypertension is not only an important component of MetS but also has similar risk factors related to metabolic diseases [11].

However, inconsistent results have been found in population-based studies between ALT and hypertension [12–14]. Some studies revealed that plasma ALT was related to blood pressures (BP) [15], and the prevalence of hypertension was obviously increased with serum ALT levels [16]. Other studies did not reveal a substantial association between serum ALT and hypertension because of small sample size [13, 17] or multiple metabolic confounding risk factors in research objects [17]. Meanwhile, a gender difference was found, the association between ALT and hypertension or BP levels was only discovered among males [18]. Therefore, no convincing evidence for the association between serum ALT levels and hypertension as well as BP existed. It is essential to investigate the possible association in a large population and fill the gap of existing research. In this study, we examined the association between serum ALT and hypertension and BP among over 21 million reproductive-aged people based on the National Free Pre-pregnancy Checkups Project (NFPCP).

## Methods

### Study design and population

This cross-sectional study was aimed to assess the association between serum ALT and hypertension as well as BP by using registration data of NFPCP from 2016 to 2017. NFPCP is a national registry project initiated by the Ministry of Finance and National Health Commission of China since 2010 and extended to 2907 counties or districts across 31 provinces in mainland China after 2013[19, 20]. The purpose of this project was providing free preconception health examination and health consultancy service for reproductive-age couples with pregnancy intentions to promote maternal and infant health in rural areas and gradually expanded to urban resident couples. Even after the expansion, the NFPCP still mainly focused on rural areas. More details about the design, organization, and implementation of NFPCP have been described previously [19]. All participants from NFPCP provided written informed consent before participation. This study was approved by the Institutional Research Review Board of the National Research Institute for Family Planning.

All participants in NFPCP between 2016 and 2017 were selected as the study population. Eligible participants were women aged 20–49 years and men aged 20–59 years with available data on BP and ALT. Participants were excluded if they have a self-reported history of chronic nephritis (*n* = 5294) or heart disease (*n* = 9465) or have taken any medication (*n* = 557,038). Participants with ALT more than 500 U/L were also excluded (*n* = 3800), due to acute liver injury, such as hepatic shock, is always accompanied by extremely high ALT levels.

### Data collection

A uniformed protocol with a standard questionnaire was used by trained staff to collect the information of participants in preconception examination, including sociodemographic characteristics, medical history, medication history, pregnancy history, family history of diseases, lifestyles, and socio-psychological factors. Sociodemographic characteristics include age, ethnicity, area, education, and occupation. Medical history covers the history of diseases, such as hypertension, heart disease, diabetes, thyroid disease, chronic nephritis, and epilepsy. Medication history contains information on medication, vaccination, and birth control measures. Pregnancy history consists of menarche age, menstrual cycle, history of pregnancy, and adverse pregnancy outcomes. Family history of diseases involves thalassemia, hemophilia, congenital heart disease, diabetes, and Down syndrome. Lifestyles comprise questions on the participant’s food preference, smoking and drinking habits, and exposure to hazardous substances at work. Socio-psychological factors include stresses from life or work, tense relationships with relatives or colleagues, financial stress, and unpreparedness for pregnancy.

Physical examinations and routine clinical laboratory examinations were performed by experienced doctors who had been trained according to standard clinical operational guidelines. Height and body weight were recorded from electronic height and weight measurement equipment for hospitals. Seated BP was measured on the right arm by physicians using an electronic sphygmomanometer in a quiet room according to the Guidelines for Physical Examination of the NFPCP. Under the notice of not drinking caffeinated beverages and keeping a light diet 3 days before the examination, a single occasion of BP measurement was taken after resting for 10 min. Blood samples after 8 h of fasting were taken and immediately stored at 4–8 °C for laboratory tests, including ALT, blood glucose, and hepatitis B surface antigen (HBsAg), within 24 h. Blood glucose was only measured among women. The specimens were tested in a local laboratory by experienced medical laboratory specialists in accordance with the National Guide to Clinical Laboratory Procedures. ALT concentration was detected using an automatic biochemical analyzer by measuring the absorbance change in nicotinamide adenine dinucleotide at 340 nm after the serum was reacted with the substrate. All procedures were conducted in local maternal and childcare service centers. After the preconception examination was completed, all data were uploaded and stored on the remote server of the NFPCP medical service information system which was developed and operated by National Research Institute for Health and Family Planning. A random sampling check was performed twice a year by The National Center of Clinical Laboratories for Quality Inspection and Detection in China to ensure the consistency of clinical results in laboratories.

### Definitions

In the current analysis, ethnicity was categorized as “Han” and “others,” area was sorted as “rural” and “urban,” and education level was classified as “below high school” and “high school or above.” Body mass index (BMI) was calculated as weight in kilograms divided by height in square meters. Participants with BMIs of < 18.5, 18.5–23.9, 24–27.9, and ≥ 28.0 kg/m^2^ were regarded as underweight, normal, overweight, and obese, respectively, based on the guideline for Chinese adults. Average salt consumption among provinces was classified as 3 groups according to per capita iodized salt consumption released by China Public Health Statistical Yearbook 2019 [21], including less consumption (Gansu, Jiangxi, Tianjin, Chongqing, Hubei, Ningxia, Guizhou, Shanxi, Fujian, and Jilin), moderate consumption (Sichuan, Heilongjiang, Hunan, Henan, Hebei, Guangxi, Zhejiang, Shaanxi, Shandong, and Shanghai), and higher consumption (Hainan, Inner Mongolia, Anhui, Guangdong, Beijing, Jiangsu, Yunnan, Qinghai, Liaoning, Sinkiang, and Tibet). Drinking status and smoking status were both classified as “Yes” or “No” as reported by the participants. Having overall stress was self-reported as having at least one of the four types of stresses: “Life or work stress,” “Tense relationship with relatives or colleagues,” “Financial stress,” or “Still not ready for pregnancy.” Fasting blood glucose was classified as normal (≤ 6.1 mmol/L or 110 mg/dl) and elevated (> 6.1 mmol/L or 110 mg/dl) levels. HBsAg status was defined as positive or negative according to HBsAg tests.

In addition to numeric ALT concentrations, ALT concentrations were classified into two levels (Group I), namely normal ALT (≤ 40 U/L) and elevated ALT (> 40 U/L) [10]. To further assess the association between different ALT levels and hypertension, ALT was also split into five categories (Group II) with an increment of 20 U/L from the lowest level (≤ 20 U/L) to the highest level (> 80 U/L) to support the analysis of its linear relationship with BP or hypertension.

Hypertension was defined as systolic blood pressure (SBP) ≥ 140 mmHg or diastolic blood pressure (DBP) ≥ 90 mmHg at seated BP measurement according to WHO diagnostic criteria [22].

### Statistical analysis

Baseline characteristics were described using mean values and standard deviation, median and interquartile range, or numbers and percentages. The *t*-tests or χ^2^ tests were performed to evaluate the differences between normotensive and hypertensive groups. The strength of associations (odds ratios [OR], and the 95% confidence interval [95% CI] of OR) between ALT and hypertension were assessed using age–sex–adjusted and multivariable-adjusted logistic regressions. Covariates in the multivariable-adjusted logistic regression included general characteristics (age, gender, ethnicity, area, education, and BMI), life behaviors (average salt consumption), history of disease (diabetes), HBsAg status, and psychological stress. In addition, the association of ALT levels and hypertension was reassessed among women after adding blood glucose into the covariates.

The age– and sex–adjusted least square means of SBP and DBP across different ALT levels were calculated to illustrate the differences of BPs among ALT levels. The associations between ALT and SBP or DBP were estimated by multivariable-adjusted linear regression in which covariates included were the same as the multivariable-adjusted logistic regression.

Subsequent statistical analyses, including subgroup analyses, interaction analyses, and sensitivity analyses, were performed to test the interaction and robustness of the association between ALT and hypertension. Subgroup analyses were used to verify the consistency of association among different populations after adjustment for multiple covariates, and then the possibility of effect modification has been tested using the Cochran-Mantel–Haenszel approach with Bonferroni-adjusted *P*-value to compare the differences of ORs for hypertension. After that, the effects of possible modifiers and ALT levels were stratified into 2 × 2 cross-tables to evaluate additive interactions by multiple indicators, including the relative excess risk due to interaction (RERI), the proportion of disease among those with both exposures that is attributable to their interaction (AP[AB]) and the synergy index (S) [23]. Multiple sensitivity analyses were also conducted to assess the robustness of the observed association between ALT and hypertension including redefined hypertension [SBP ≥ 130 mmHg or DBP ≥ 80 mmHg] in logistic regression models by using the new classification recommended of American Heart Association in 2017 [24] and removing important modifiers in the multivariable-adjusted logistic regression model in turn. Lastly, the shape of the association between ALT and hypertension was presented by multivariable-adjusted restricted cubic spline models with “rms” package(https://cran.r-project.org/web/packages/rms). All analyses were performed in R software 3.5.0.

## Results

**Fig. 1:**
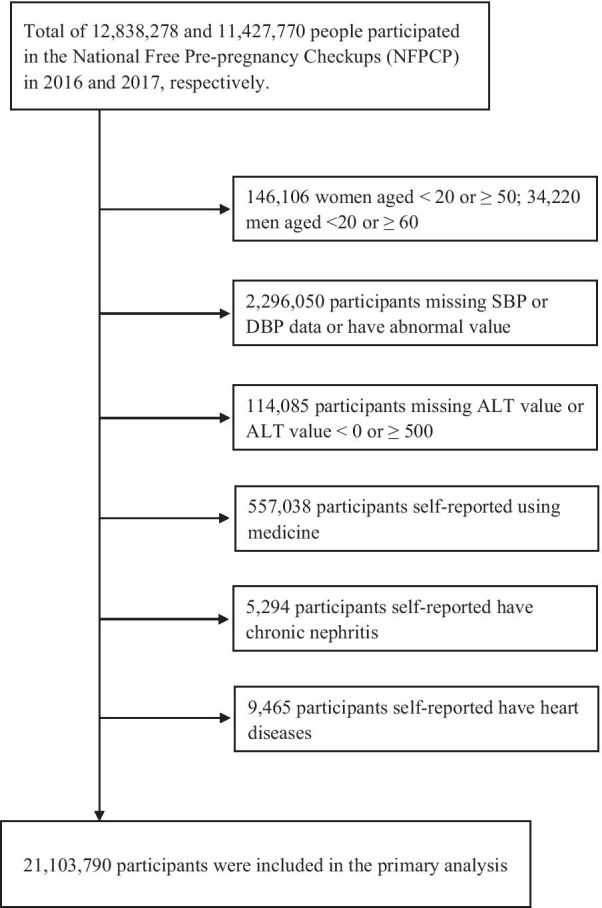
Flowchart of the study population in the association between serum alanine aminotransferase and hypertension

Overall, 24,085,722 people, including 11,986,918 women aged 20–49 years and 12,098,804 men aged 20–59 years, participated in NFPCP in 2016–2017 nationwide. Finally, 21,103,790 participants, including 10,095,225 men (47.84%) and 11,008,565 women (52.16%), were eligible for this cross-sectional study. The flowchart of participant recruitment is provided in Fig. 1.

The average age of the enrolled population was 29.55 ± 6.19 years old. Most of the participants belong to the Han ethnicity (87.98%) and came from rural areas (86.95%). The prevalence of hypertension was 4.21% (887,509 out of 21,103,790) in the total population, 2.59% (285,455 out of 11,008,565) in women, and 5.96% (602,054 out of 10,095,225) in men. Compared with normotensive participants, people with hypertension were more likely to be older; less educated; with higher salt consumption, frequent alcohol drinking, and heavy smoking; have higher BMI, ALT, and blood glucose concentrations; and suffered from diabetes and psychological stress (Table 1).

**Table 1:** Demographic characteristics of participants according to hypertensive status

Variables	Total	Normotensive	Hypertensive	P*
	(*n* = 21,103,790)	(*n* = 20,216,281)	(*n* = 887,509)	
Gender, Female [*n*(%)]^†^	11,008,565 (52.16)	10,723,110 (53.04)	285,455 (32.16)	< 0.001
Age, *y* (Mean ± SD)^††^	29.55 ± 6.19	29.39 ± 6.10	33.03 ± 7.16	< 0.001
Ethnicity, Han [*n* (%)]	18,567,773 (87.98)	17,781,209 (87.95)	786,564 (88.63)	0.171
Area, Rural [*n* (%)]	18,348,932 (86.95)	17,625,871 (87.19)	723,061 (81.47)	< 0.001
Region, South [*n* (%)]	12,248,880 (58.04)	11,719,810 (95.68)	529,070 (4.32)	< 0.001
Education, Below High School [*n* (%)]	12,139,927 (57.52)	11,626,869 (57.51)	513,058 (57.81)	< 0.001
Drinking, Yes [*n* (%)]	3,033,405 (14.39)	2,791,062 (13.82)	242,343 (27.33)	< 0.001
Smoking, Yes [*n* (%)]	2,816,211 (13.36)	2,614,132 (12.94)	202,079 (22.79)	< 0.001
Salt consumption, Excessive [*n* (%)]	5,416,550 (25.67)	5,125,161 (25.57)	291,389 (33.21)	< 0.001
BMI, kg/m^2^ (Mean ± SD)	22.63 ± 3.32	22.52 ± 3.25	25.04 ± 4.06	< 0.001
< 18.5 [*n* (%)]	1,558,090 (7.38)	1,533,888 (7.59)	24,202 (2.73)	
18.5–23.9 [*n* (%)]	13,382,798 (63.41)	13,029,173 (64.45)	353,625 (39.84)	
24.0–27.9 [*n* (%)]	4,798,023 (22.74)	4,477,431 (22.15)	320,592 (36.12)	
≥ 28 [*n* (%)]	1,327,226 (6.29)	1,140,466 (5.64)	186,760 (21.04)	
Heart Rate, *bp* (Mean ± SD)	76.02 ± 7.76	75.85 ± 7.52	79.80 ± 11.32	< 0.001
ALT, *U/L* (Median, IQR)	20.00,15.70	20.00, 15.00	26.00, 16.80	< 0.001
Blood glucose, mmol/L (Mean ± SD)^§^	4.93 ± 1.00	4.92 ± 0.99	5.21 ± 1.42	< 0.001
HBsAg, Positive [*n* (%)]	1,208,067 (5.73)	1,154,190 (5.71)	53,877 (6.08)	< 0.001
History of Diabetes, Yes [*n* (%)]	10,047 (0.05)	8,210 (0.04)	1,837 (0.21)	< 0.001
Overall stress, Yes [*n* (%)]	2,411,365 (11.47)	2,271,218 (11.27)	140,147 (15.85)	< 0.001

SD: standard deviation; BMI: body mass index; IQR: interquartile range; HBsAg: hepatitis B surface antigen

*Age and Sex adjusted *P*-value was calculated using Logistic Regression Model

^†^Age adjusted *P-*value was calculated using Logistic Regression Model

^††^Sex adjusted *P-*value was calculated using Logistic Regression Model

^§^Blood glucose was only measured among women

**Fig. 2:**
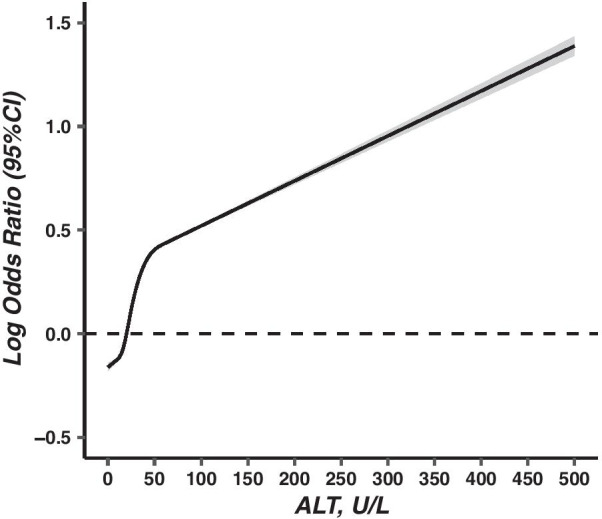
Associations between serum alanine aminotransferase and hypertension (OR, 95% CI). Abbreviations: OR: odds ratios; CI: confidence interval. Hypertension defined by SBP ≥ 140 mmHg or DBP ≥ 90 mmHg. Serum alanine aminotransferase was modelled with a restricted cubic spline model. The vertical axis is on a log scale. Covariates included age, sex, ethnicity, area, education, body mass index, average salt consumption, history of diabetes, overall pressure and hepatitis B surface antigen status. The dash line represents the reference level. Imaging packages: “rms”, “ggplot2”

The median ALT concentration was 20.00 U/L (IQR: 15.70) among the total population and significantly higher (*W* = 67,846, *P* < 0.001) in the hypertensive group (26.00 U/L, IQR: 22.80) than in the normotensive group (20.00 U/L, IQR: 15.00). According to the standardized β value in the multivariable-adjusted logistic regression model, the specific contribution of continuous ALT to hypertension ranked fifth among 11 common cardiovascular risk factors after BMI, age, excessive salt consumption, and gender. The association between continuous ALT concentrations and hypertension odds was essentially linear (*t* = 176.92, *P* < 0.001, Fig. 2). With each increase of ALT concentration (1U/L), the odds of hypertension increased by 1% (OR = 1.01; 95% CI = 1.01–1.01) (Table 2).

**Table 2:** Association between alanine aminotransferase levels and hypertension

ALT levels (U/L)	Hypertensive *n*, (%)	Age-Sex-adjusted OR (95% CI)	Multivariable-adjusted OR (95% CI)*
Continuous variable			
ALT	887,509 (4.21)	1.01 (1.01,1.01)	1.01 (1.01,1.01)
Categorical variable			
Group I			
0–40	676,607 (3.63)	1	1
> 40	210,902 (8.56)	2.05 (2.04,2.07)	1.55 (1.54,1.56)
Group II			
0–20	308,328 (2.87)	1	1
20.01–40	368,279 (4.65)	1.35 (1.34,1.35)	1.22 (1.21,1.22)
40.01–60	112,137 (8.02)	2.21 (2.20,2.23)	1.67 (1.65,1.68)
60.01–80	47,326 (8.88)	2.54 (2.52,2.57)	1.78 (1.76,1.80)
> 80	51,439 (9.64)	2.90 (2.88,2.93)	1.92 (1.90,1.94)
*P*_*for trend*_^†^		< 0.001	< 0.001

ALT: alanine aminotransferase; OR: odds ratios; CI: confidence interval;

*Adjusted for age, sex, ethnicity, area, education, body mass index, average salt consumption, history of diabetes, overall stress and hepatitis B surface antigen status

^†^P for trend was calculated by setting categorized ALT levels as a continuous variable

Moreover, as a categorized variable, a total of 2,463,734 people had elevated ALT level (> 40 U/L), which accounted for 11.67% of all participants. The prevalence of hypertension was 3.63% (95% CI = 3.62–3.64) and 8.56% (95% CI = 8.52–8.60) in people with normal and elevated ALT levels, respectively. The multivariable-adjusted OR of hypertension was 1.55 (95% CI = 1.54–1.56) in participants with elevated ALT level compared with normal ALT level. In addition, the multivariable- adjusted ORs for hypertension were 1, 1.22 (1.21, 1.22), 1.67 (1.65, 1.68), 1.78 (1.76, 1.80), and 1.92 (1.90, 1.94) in participants with ALT levels of ≤ 20, 20.01–40, 40.01–60, 60.01–80, and > 80 U/L, respectively (*t* = 192.02, *P *_*for trend*_ < 0.001) (Table 2). The association between ALT levels and hypertension did not changed after adding blood glucose as covariate into the multivariable-adjusted logistic regression model among women (Table 5 of Appendix).

ALT concentration is linearly associated with SBP (*t* = 149.46, *P* < 0.001) and DBP (*t* = 163.35, *P* < 0.001). The mean values of SBP and DBP after age- sex-adjusted least-squares estimation remarkably rose with increasing ALT levels (Table 3). The average SBP and DBP rose by 1.19 and 0.88 mmHg respectively in people with elevated ALT level compared with normal ALT after multivariable adjustment (Table 3). Furthermore, SBP and DBP rose by 1.83 and 1.20 mmHg in each 20 U/L increments in ALT concentration in Group II (Table 6 of Appendix). Similar trends were also shown in hypertensive and normotensive populations.

**Table 3:** Associations between alanine aminotransferase levels and systolic or diastolic blood pressures

ALT Levels (U/L)	Total				Hypertensive				Normotensive			
	LS-Means*	S.E*	β^†^	*P* value^†^	LS-Means*	S.E*	β^†^	*P* value^†^	LS-Means*	S.E*	β^†^	*P* value^†^
*SBP*												
ALT, continuous	113.98	0.00	0.02	< 0.001	137.57	0.02	0.02	< 0.001	112.95	0.00	0.01	< 0.001
*Group I*												
0–40	113.50	0.00	–	–	136.73	0.02	–	–	112.61	0.00	–	–
> 40	117.66	0.01	1.19	< 0.001	140.30	0.03	1.31	< 0.001	115.63	0.01	0.57	< 0.001
*Group II*												
0–20	112.57	0.00	–	–	135.95	0.03	–	–	111.83	0.00	–	–
20.01–40	114.75	0.00	0.27	< 0.001	137.37	0.03	-0.01	0.905	113.68	0.00	0.16	< 0.001
40.01–60	117.27	0.01	1.22	< 0.001	139.72	0.05	1.1	< 0.001	115.41	0.01	0.63	< 0.001
60.0–80	117.91	0.02	1.39	< 0.001	140.57	0.07	1.47	< 0.001	115.79	0.01	0.66	< 0.001
> 80.01	118.45	0.02	1.59	< 0.001	141.30	0.07	1.64	< 0.001	116.09	0.01	0.71	< 0.001
*P*_*for trend*_^††^	< 0.001				< 0.001				< 0.001			
*DBP*												
ALT, continuous	73.88	0.00	0.01	< 0.001	93.41	0.01	0.01	< 0.001	73.02	0.00	0.01	< 0.001
*Group I*												
0–40	73.57	0.00	–	–	93.27	0.01	–	–	72.82	0.00	–	–
> 40	76.20	0.01	0.88	< 0.001	93.86	0.03	0.37	< 0.001	74.60	0.01	0.41	< 0.001
*Group II*												
0–20	72.92	0.00	–	–	93.11	0.02	–	–	72.29	0.00	–	–
20.01–40	74.45	0.00	0.39	< 0.001	93.41	0.02	0.32	< 0.001	73.55	0.00	0.28	< 0.001
40.01–60	75.93	0.01	0.98	< 0.001	93.64	0.03	0.42	< 0.001	74.45	0.01	0.52	< 0.001
60.0–80	76.38	0.01	1.15	< 0.001	94.05	0.05	0.75	< 0.001	74.71	0.01	0.57	< 0.001
> 80	76.74	0.01	1.31	< 0.001	94.18	0.05	0.77	< 0.001	74.89	0.01	0.63	< 0.001
*P*_*for trend*_^††^	< 0.001				< 0.001				< 0.001			

SBP: systolic blood pressure; DBP: diastolic blood pressure; ALT: alanine aminotransferase; LS-Means: Least-squares means; S.E: standard error of the LS-Means

*Adjusted for age

^†^Adjusted for age, sex, ethnicity, area, education, body mass index, average salt consumption, history of diabetes, overall stress and hepatitis B surface antigen status

^††^P for trend were calculated by setting categorized ALT levels as a continuous variable

**Fig. 3:**
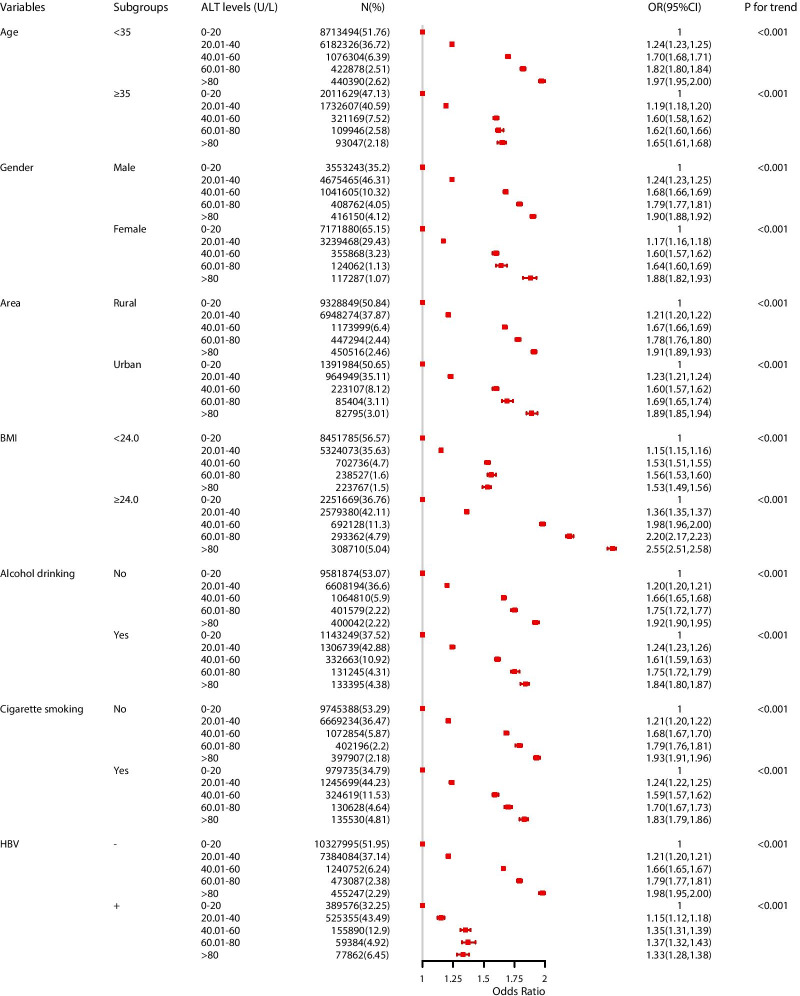
Association between serum alanine aminotransferase and hypertension among subgroups (OR, 95% CI). Abbreviations: OR: odds ratios; CI: confidence interval; BMI: body mass index; ALT: alanine aminotransferase; HBsAg: hepatitis B surface antigen. Multi-adjusted OR: odds ratio in multivariable logistic regression. Covariates in subgroup analyses were the same with variables that adjusted in multivariable logistic regression model among total participants, except for the grouping variable. Imaging packages: “forestplot”, “ggplot2”

Subgroup analyses showed that the odds for hypertension were increased along with categorized ALT levels across all subgroups (*P *_*for trend*_ < 0.001, Fig. 3). Besides, the strength of association was modified by BMI, alcohol drinking, cigarette smoking and HBsAg status (*χ*^*2*^ = 52,228, 100,730, 105,347, and 131,220, respectively. *P *_*Cochran-Mantel–Haenszel*_ < 0.001). People with elevated ALT level accompanied by higher BMI (≥ 24 kg/m^2^), alcohol drinking or cigarette smoking had positive additive interactions (RERI > 0, S > 1) and significantly increased their odds for hypertension (Table 4). People who have positive HBsAg status had negative additive interactions (RERI < 0, S < 1) in the association between ALT levels and hypertension, although the odds for hypertension was also increased 0.22 times (OR:1.22, 95% CI = 1.20–1.24) (Table 4). Additionally, the association between ALT and hypertension did not substantially change by using multiple sensitivity analyses (Tables 7 and 8 of Appendix).

**Table 4:** Addictive interaction of important modifiers on the association between alanine aminotransferase levels and hypertension

Variables	*n* (%)	Crude OR (95% CI)	Multi-adjusted OR (95% CI)	RERI	AP[AB]	S
ALT & BMI*				1.393	0.322	1.720
0–40 & < 24	13,775,858 (65.28)	1	1			
0–40 & ≥ 24	4,831,049 (22.89)	3.12 (3.10,3.13)	2.47 (2.46,2.49)			
> 40 & < 24	1,165,030 (5.52)	1.71 (1.69,1.73)	1.46 (1.45,1.47)			
> 40 & ≥ 24	1,294,200 (6.13)	5.87 (5.83,5.91)	4.23 (4.30,4.36)			
ALT & Drinking^†^				0.214	0.093	0.377
0–40 & No	16,181,008 (76.67)	1	1			
0–40 & Yes	2,439,518 (11.56)	2.23 (2.22,2.25)	1.54 (1.53,1.55)			
> 40 & No	1,867,240 (8.85)	2.37 (2.36,2.39)	1.55 (1.54,1.56)			
> 40 & Yes	593,887 (2.81)	4.57 (4.53,4.60)	2.31 (2.29,2.33)			
ALT & Smoking^†^				0.014	0.078	1.195
0–40 & No	16,398,611 (77.70)	1	1			
0–40 & Yes	2,225,434 (10.55)	1.90 (1.89,1.92)	1.27 (1.26,1.28)			
> 40 & No	1,870,788 (8.86)	2.45 (2.44,2.47)	1.57 (1.56,1.58)			
> 40 & Yes	590,777 (2.80)	3.73 (3.69,3.76)	1.86 (1.84,1.88)			
ALT & HBsAg^††^				-0.362	-0.297	1.017
0–40 & −	17,712,079 (83.93)	1	1			
0–40 & +	914,931 (4.34)	1.07 (1.06,1.08)	1.00 (0.98,1.01)			
> 40 & −	2,169,086 (10.28)	2.59 (2.58,2.61)	1.58 (1.58,1.59)			
> 40 & +	293,136 (1.39)	1.80 (1.77,1.83)	1.22 (1.20,1.24)			

ALT: alanine aminotransferase; OR: odds ratio; BMI: body mass index; HBsAg: hepatitis B surface antigen; RERI: the relative excess risk due to interaction; AP[AB]: the proportion of disease among those with both exposures that is attributable to their interaction; S: the synergy index

*n*(%): n, number of participants; %, proportion of participants

*Adjusted for age, sex, ethnicity, area, education, average salt consumption, history of diabetes, overall stress and hepatitis B surface antigen status

^†^Adjusted for age, sex, ethnicity, area, education, body mass index, average salt consumption, history of diabetes, overall stress and hepatitis B surface antigen status

^††^Adjusted for age, sex, ethnicity, area, education, body mass index, average salt consumption, history of diabetes and overall stress

**Table 5:** Association between ALT levels and hypertension prevalence among women

ALT levels (U/L)	Hypertensive, n (%)	Age-adjusted OR (95% CI)	Model I	Model II
			Multi-adjusted OR (95% CI)	Multi-adjusted OR (95% CI)
*Group I*				
0–40	257,764 (2.48)	1	1	1
40.01–	27,691 (4.64)	1.91 (1.88,1.93)	1.56 (1.54,1.58)	1.52 (1.50,1.54)
*Group II*				
0–20	162,779 (2.27)	1	1	1
20.01–40	94,985 (2.93)	1.27 (1.26,1.28)	1.17 (1.16,1.18)	1.17 (1.16,1.18)
40.01–60	15,838 (4.45)	1.94 (1.91,1.97)	1.59 (1.57,1.62)	1.56 (1.53,1.58)
60.01–80	5730 (4.62)	2.08 (2.02,2.14)	1.64 (1.60,1.69)	1.59 (1.55,1.64)
80.01–	6123 (5.22)	2.46 (2.40,2.53)	1.87 (1.82,1.93)	1.79 (1.74,1.84)
P for trend^†^		< 0.001	< 0.001	< 0.001

ALT: alanine aminotransferase; OR: odds ratios; CI: confidence interval

Model I: Adjusted for age, sex, ethnicity, area, education, body mass index, average salt consumption, history of diabetes, overall pressure and hepatitis B surface antigen status

Model II: add fasting blood glucose concentration as a new covariate based on Model I

^†^P for trend were calculated by setting categorized ALT levels as a continuous variable

**Table 6:** Average rising of SBP and DBP after age-adjusted least squares estimation

ALT Classification	Total		Hypertensive		Normotensive	
	LS-Means*	S.E	LS-Means*	S.E	LS-Means*	S.E
*SBP*						
Group I	4.160	0.007	3.570	0.038	3.024	0.007
Group II	1.831	0.003	1.496	0.015	1.401	0.002
*DBP*						
Group I	2.626	0.006	0.593	0.029	1.778	0.005
Group II	1.200	0.002	0.279	0.011	0.873	0.002

SBP: systolic blood pressure; DBP: diastolic blood pressure; ALT: alanine aminotransferase; LS-Means: Least-squares means; SD: standard deviation;

*Adjusted for age

**Table 7:** Sensitive analysis by using new classification of hypertension (SBP > 130|DBP > 80)

ALT levels (U/L)	Hypertensive, *n* (%)	Age-Sex-adjusted OR (95% CI)	Multi-adjusted OR (95% CI)*
*Group I*			
0–40	5,753,021 (30.86)	1	1
40.01–	1,091,213 (44.29)	1.37 (1.36,1.37)	1.17 (1.16,1.17)
*Group II*			
0–20	2,935,226 (27.37)	1	1
20.01–40	2,817,795 (35.60)	1.16 (1.16,1.16)	1.09 (1.08,1.09)
40.01–60	605,968 (43.36)	1.42 (1.41,1.43)	1.21 (1.20,1.21)
60.01–80	239,252 (44.90)	1.51 (1.50,1.52)	1.22 (1.22,1.23)
80.01–	245,993 (46.11)	1.60 (1.59,1.61)	1.25 (1.25,1.26)
*P *_*for trend*_^†^		< 0.001	< 0.001

ALT: alanine aminotransferase; OR: odds ratios; CI: confidence interval;

^*^Adjusted for age, sex, ethnicity, area, education, body mass index, average salt consumption, history of diabetes, overall pressure and hepatitis B surface antigen status;

^†^P for trend were calculated by set categorized ALT levels as a continuous variable

**Table 8:** Sensitivity analysis by excluding important modifiers in the multivariable-variable adjusted model (OR, 95%CI)

ALT levels (U/L)	Total Model^*^	Excluding Variables		
		Age	BMI	HBsAg
*Group I*				
0–40	1	1	1	1
40.01–	1.55 (1.54,1.56)	1.44 (1.43,1.45)	2.01 (2,2.02)	1.52 (1.51,1.53)
*Group II*				
0–20	1	1	1	1
20.01–40	1.22 (1.21,1.22)	1.23 (1.22,1.23)	1.37 (1.36,1.38)	1.21 (1.20,1.22)
40.01–60	1.67 (1.65,1.68)	1.61 (1.59,1.62)	2.19 (2.17,2.20)	1.63 (1.62,1.64)
60.01–80	1.78 (1.76,1.80)	1.64 (1.62,1.66)	2.51 (2.48,2.53)	1.74 (1.72,1.76)
80.01–	1.92 (1.90,1.94)	1.68 (1.66,1.70)	2.88 (2.85,2.91)	1.87 (1.85,1.89)

BMI: body mass index; ALT: alanine aminotransferase; HBsAg: hepatitis B surface antigen;

^*^Multi-variable Model: Adjusted for age, sex, ethnicity, area, education, body mass index, average salt consumption, history of diabetes, overall pressure and hepatitis B surface antigen status

## Discussion

To our knowledge, our study is the first to examine the association between ALT and hypertension as well as BP in reproductive-aged adults excluding any medication, with a large sample size of more than 21 million people. Our study adds new information to the public that liver health may be associated with BP.

Conventional risk factors were all found to be related to hypertension in this study. The attributable contribution of continuous ALT elevation toward hypertension was just below BMI, age, excessive salt consumption and gender in multivariable-adjusted logistic model, which suggested that elevated ALT might be a risk factor for hypertension. The odds for hypertension was increased by 55% among people with elevated ALT compared with those who have normal ALT. This finding was similar with previous evidence that elevated ALT increased the risks for hypertension [25], MetS [7] and CVD [26].

Our results revealed a linear and strong association not only between ALT levels and hypertension odds but also between ALT levels and SBP or DBP. Besides, the positive trend between ALT levels and BP in the hypertensive and normotensive subgroups suggested that the association between serum ALT and BP was coherent and that the SBP and DBP were increasing along with serum ALT elevating whether in hypertensive or normotensive populations. These findings resulted from the hypothesis that serum ALT concentration may associate with blood pressures in the light of evidence that serum ALT was demonstrated to be associated with MetS [5–7]. Reliable prospective cohort studies are warranted to confirm this possible relationship observed in our study and further disclose the causal relationship between serum ALT concentrations elevation and hypertension.

Although some previous studies only reported a remarkable association between ALT and hypertension in people with certain demographic characteristics [17, 27], we found a similar association in subgroups, including BMI, HBsAg, sociodemographic characteristics (age, gender, and area) and health-related behaviors (alcohol drinking and cigarette smoking). Furthermore, interaction effects were also found between serum ALT levels and some conventional hypertensive risk factors. People with elevated ALT accompanied by BMI ≥ 24 kg/m^2^, alcohol consumption or cigarette smoking significantly increased their odds for hypertension than those with only elevated ALT level. Few existing studies have reported the interaction effects of related confounding risk factors toward the association between ALT and hypertension. A previous study has reported that the association between ALT and hypertensive risks is only found in people with central obesity [5]; excessive alcohol is a crucial cause of ALT elevation [28], rather than short-term or light alcohol consumption [29]; moreover, the additional effects between cigarette smoking and elevated serum ALT with respect to hypertension have not been discussed. In this study, we found that overweight or obesity, alcohol drinking, and cigarette smoking can further increase the odds for hypertension among people with elevated ALT levels. More studies are needed to confirm these findings and people with serum ALT elevation should pay attention to higher BMI levels, alcohol drinking and cigarette smoking. Interestingly, the odds for hypertension was higher in the negative HBsAg group than in the positive HBsAg group within elevated ALT in our analyses, which was consistent with findings that HBsAg-positive population has a lower odds for hypertension compared with those with a negative status [30].

A growing body of evidence strongly suggested that serum ALT concentration, the most appropriable predictor of liver fat accumulation [31] and metabolic derangement [32], is related to the pathophysiologic features of increasing BP. Aminotransferases are ubiquitous enzymes that catalyze the reversible transfer of amino groups from amino acids to α-keto acids and play a vital role in amino acid metabolism [33]. Elevated serum ALT levels are independently associated with a higher prevalence of dyslipidemia [34]; increasing levels of serum triglyceride contents [35]; and higher low-density lipoprotein cholesterol, non-high-density lipoprotein (HDL) cholesterol, and lower HDL cholesterol [36]. Moreover, serum ALT is closely related to glucose tolerance, insulin resistance and other conventional risk factors of metabolic diseases [6]. Higher ALT levels are also substantially correlated with excessive inflammation and oxidative stress [37]. In addition, elevated ALT concentration is positively associated with faster heart rate [6], higher risk of carotid atherosclerosis [38], increased arterial stiffness [39] and can behave as a surrogate marker of left ventricular hypertrophy and carotid artery changes [40]. All these features mentioned above contribute to the development of hypertension. Prospective studies still need to verify the pathophysiologic mechanisms between serum ALT and hypertension.

To date, our study is the largest nationwide epidemiological study that investigated the association between serum ALT without limitation of the clinical reference range and the odds of hypertension in over 21 million people. The large sample size can ensure sufficient statistical power of all analyses. Possible confounding effects regarding sociodemographic characteristics, lifestyle behaviors, psychological stresses, and history of diseases were considered in statistical analyses. Strict inclusion and exclusion criteria were adopted, in which people who were taking medicines were excluded to avoid the adverse influence of medications on ALT and blood pressures. In addition, we conducted several sensitivity analyses to verify the robustness of the observed association. Above all, the participants recruited in NFPCP have specific demographic characteristics, such as having an explicit wish to conceive, being younger, and more concerned about healthy lifestyles than the general population. The specified younger and healthier participants enabled us to explore the natural association between ALT and hypertension, in which ALT and BP were not influenced by medication-, age-, and behavior-related changes.

However, several limitations also existed. First, a cross-sectional design would not draw a causal relationship between serum ALT and hypertension. Prospective studies based on large-scale populations are needed to verify the authenticity of the observed association between serum ALT and BPs as well as the odds for hypertension. Second, not all the reproductive-aged people who planned to conceive in 2016–2017 had participated in the NFPCP in China, and most of the included participants were from rural areas (86.95%). Thus, the hypertension prevalence in our study may have been underestimated. Third, a misclassification bias of hypertension could be introduced because only one single seated BP was measured after resting for 10 min, despite the fact that the participants were informed to not drink caffeinated beverages, have a good sleep and keep a light diet 3 days before the examination. Fourth, ALT concentration may also be affected by many lifestyle behaviors a few days before examination, such as sleeping, diet and drinking status. Fifth, many questions were self-reported according to the structured questionnaire, including life behaviors and history of disease, which may introduce recall bias. Sixth, no other liver enzyme was available in NFPCP laboratory tests to support the observed association between ALT and hypertension. Finally, our research is mainly carried out in people who are relatively young and take participate in NFPCP. The findings of this research may be helpful for promoting the health of expectant parents but may not be applicable to other populations with older age.

## Conclusions

Findings from this study showed a strong, independent, and linear association between elevated serum ALT and hypertension, which indicated that abnormal liver metabolism marked elevated serum ALT may correlate with the odds for hypertension. Future prospective cohort studies are warranted to confirm the possible relationship between ALT concentrations and hypertension and further disclose the underlying mechanisms.

## Data Availability

The NFPCP data contained private sensitive data, according to the governance restrictions, these data were forbidden to share with the public in order protect individual data confidentiality. Data are available from the corresponding authors upon reasonable request and with permission of National Research Institute for Family Planning in China.

## Appendix

See Tables 5, 6, 7 and 8.

## Abbreviations

ALT: Alanine aminotransferase
BP: Blood pressure
OR: Odds ratio
BMI: Body mass index
MetS: Metabolic syndrome
CVD: Cardiovascular disease
NFPCP: National Free Pre-pregnancy Checkups Project
HBsAg: Hepatitis B surface antigen
SBP: Systolic blood pressure
DBP: Diastolic blood pressure
WHO: Word Health Organization
95% CI: 95% Confidence interval
